# Preclinical cardiovascular changes in children with obesity: A real-time 3-dimensional speckle tracking imaging study

**DOI:** 10.1371/journal.pone.0205177

**Published:** 2018-10-11

**Authors:** Chunquan Zhang, Yiwen Deng, Yanna Liu, Yan Xu, Yanling Liu, Li Zhang, Xiongwen Chen, Mingxing Xie, Shuping Ge

**Affiliations:** 1 Department of Ultrasound, The Second Affiliated Hospital of Nanchang University, Nanchang, China; 2 Department of Pediatrics, The Second Affiliated Hospital of Nanchang University, Nanchang, China; 3 Department of Ultrasound, Union Hospital, Tongji Medical College of Huazhong University of Science and Technology, Wuhan, China; 4 Department of Physiology, Temple University School of Medicine, Philadelphia, PA, United States of America; 5 The Heart Center, St. Christopher's Hospital for Children and Drexel University College of Medicine, Philadelphia, PA, United States of America; University of Cincinnati College of Medicine, UNITED STATES

## Abstract

The aims of this study were (1) to quantify changes in 3-dimensional (3D) strain in obese children using real-time 3D echocardiography (RT3DE) and 3D speckle tracking echocardiography (3DSTE), and (2) to investigate the utility of left ventricular (LV) strain variables in measuring early cardiovascular changes in children with obesity. A total of 181 obese children (study group) aged 4–18 years old were prospectively enrolled and compared with 229 healthy subjects (control group). We acquired demographic, clinical, biochemical, and 2D echocardiography/Doppler data. Also, RT3DE and 3DSTE were performed to measure LV volume, left ventricular ejection fraction (LVEF), LV mass (LVM), LV peak systolic global longitudinal strain (GLS), radial strain (GRS), circumferential strain (GCS), and global strain (GS). There were significant differences in anthropometric measurements, blood pressures, Cholesterol, C-reactive protein (CRP), Intima-media thickness (IMT), left atrium end-systolic dimension (LASD), interventricular septal end-diastolic dimension (IVSD), LV posterior wall end-diastolic dimension (LVPWD), LV end-diastolic dimension (LVEDD), LV end-systolic dimension (LVESD), LV end-diastolic volumes (LVEDV), and LV end-systolic volumes (LVESV), E and A velocities, E/A,e’, e’/a’, E/e’, LVM, LV mass index (LVMI), GLS, GRS, GCS, and GS between the study and control groups. The receiver operating characteristic curves (ROC) for the statistically significant echocardiographic variables showed that the range of areas of ROC curves varied from 0.76 (GLS), 0.74 (GRS), 0.72 (LASD), to 0.58 (LVESD), respectively. In conclusion, LV 3D strain variables by RT3DE and 3DSTE decrease in obese children. LV 3D strain is more sensitive than other echocardiographic and vascular ultrasound variables in detecting cardiovascular changes in children with obesity.

## Introduction

The prevalence of obesity has increased significantly in children and adolescents and has become a major risk factor for cardiovascular disease (CVD) [1–3]. It was reported that 60%-80% children with obesity would develop into adult obesity which is associated with left ventricle (LV) dilation, increased wall stress, hypertrophy, and heart failure [2,3]. In addition, obesity is associated with other risk factors, e.g., dyslipidemia, hypertension, glucose tolerance, inflammatory state, obstructive sleep apnea [4]. There is growing evidence that many of these cardiovascular changes occur in childhood and adolescence [5–9]. Identification of these risk factors is important because early prevention (primordial prevention) or treatment (primary prevention) to reverse the risk factors is most likely to be more effective [10].

Among the various co-morbidities, LV dysfunction remains the most significant cause of morbidity and mortality [2]. In addition to traditional LV functional measurements, e.g. LV ejection fraction, myocardial velocities determined by tissue doppler imaging (TDI) do not rely on geometric assumptions but are inherently unidimensional, angle-dependent, variable with age, and influenced by anthropometrics and heart rate [11,12]. Using both echocardiography and cardiac magnetic resonance imaging, myocardial strain has been shown to be more robust for assessment of regional ventricular myocardial function [13–17]. Currently, two-dimensional (2D) and three-dimensional (3D) speckle tracking echocardiography (STE) have been introduced to improve the accuracy of quantifying myocardial strain. Comparing with 2DSTE, 3DSTE has no geometrical assumption, unaffected by foreshortening of the LV, and more accurate and reproducible in patients with good image quality [18].

We have previously validated the feasibility, reproducibility and normal ranges of 3DSTE in children [19,20]. The aims of this study were (1) to quantify alterations in 3D strain in children with obesity using real-time 3D echocardiography and 3DSTE; and (2) to investigate the utility of LV strain variables in measurement of early cardiovascular changes in children with obesity.

## Methods

### Study population

The study was conducted prospectively in the 2^nd^ Affiliated Hospital of Nanchang University in Nanchang, Jiangxi Province, China from March 2015 to October 2017. A total of 181 obese children and 229 healthy children who met the inclusion criteria were enrolled. Obese children were recruited from pediatric clinic and healthy children were recruited from department of physical examination.

Inclusion criteria: (1) Study group: children with BMI more than the 95^th^ percentile for age and gender, respectively; (2) Control group: children with BMI less than the 85^th^ percentile for age and gender, respectively (for both groups, the BMI reference values for the Chinese pediatric population [21] were used); (3) no CVD, determined by history, physical examination and conventional echocardiography; (4) consent was obtained.

Exclusion criteria: children with (1) secondary cause of obesity (such as known genetic syndromes); (2) structural heart disease; (3) other significant diseases, including hypertension, diabetes mellitus, thyroid disorder, renal disorder, autoimmune disease, and sleep apnea; (4) children taking any prescription medications; (5) poor echocardiographic windows and image quality, object with missing data.

The BMI of the children was calculated as BMI = body weight (kg)/height (m)^2^.

The research protocol was approved by the Ethics Committee of the 2^nd^ Affiliated Hospital of Nanchang University. Written informed consent was obtained from parents or guardians before enrollment.

### Clinical data

Demographic and clinical data were obtained during the initial visit. Anthropometric measurements, including weight and height, were obtained and the BMI was calculated. Height was measured by wall-mounted stadiometers, weight by balance beam scales, waist circumference (WC) as the narrowest circumference between the margin of the lower rib and anterior superior iliac crest and hip circumference (HC) as the maximum circumference at the level of the buttock. Arterial blood pressure was measured using the right upper arm after a 10-minute rest in the supine position in a quiet room using calibrated cuff sphygmomanometers with appropriate cuff size [22]. Ultrasound measurements of subcutaneous fat thickness were taken at the posterior side of the upper arm above the triceps brachii.

### Laboratory blood biochemistry data

Blood samples were collected from study subjects in the morning after an overnight fast and transferred to the laboratory immediately for analysis from March 2015 to October 2017. A fasting lipid panel was determined with the Friedewald equation. Fasting glucose levels were measured in a fluoride-oxalate sample. C-reactive protein (CRP) levels were measured with a high-sensitivity, double-antibody sandwich enzyme-linked immunosorbent assay. Plasma homocysteine levels were determined with a modified automated assay [23].

### Conventional echocardiographic assessment

All conventional echocardiographic studies were performed using a Philips IE33 ultrasound system (Philips Medical System, Bothell, WA, USA) based on the guidelines of the American Society of Echocardiography [18]. All images were recorded and stored digitally for offline analysis. Measurements of the left ventricular end-diastolic dimension (LVEDD) and left ventricular end-systolic dimension (LVESD) were acquired in the parasternal long-axis view obtained perpendicular to the LV long axis and measured at the level of the mitral valve leaflet tips, at the end of LV diastole and systole, respectively. Interventricular septal end-diastolic dimension (IVSD) and left ventricular posterior wall end-diastolic dimension (LVPWD) were measured in the basal ventricular segment of the respective myocardial wall at the end of diastole. Left atrium end-systolic dimension (LASD) was measured in the parasternal long-axis view perpendicular to the aortic root long axis, at the level of the aortic sinuses by using the leading-edge to leading-edge convention. In the apical 4-chamber view, early (E) and late (A) transmitral inflow velocities were determined using conventional pulsed-wave spectral Doppler echocardiography. Early (e′) and late (a′) diastolic mitral annular peak velocities were measured by pulsed-wave spectral TDI from the same view, on the septal side of the mitral annulus. All the measurements were repeated three times, and the average was calculated.

### Vascular measurements

All the vascular measurements were performed with a 3–11 MHz linear-array transducer and the Philips IE33 system. Brachial artery flow-mediated dilation (FMD) was measured to assess endothelial function. The subjects were in the supine position. The dimension of the brachial artery was measured by calculating the distance between the proximal and distal intima (D1) during diastoles. Ischemia was caused for five minutes and the artery measurement was repeated 60 seconds after ending the compression (D2) during diastoles. The FMD (%) was calculated through the equation: (D2-D1)/D1 × 100% [24]. Measurements of carotid artery intima-media thickness (IMT), the end-systolic dimension (Ds) and the end-diastolic dimension (Dd) of the common carotid artery were taken on the far wall of the common carotid artery 10 mm proximal to the bifurcation [25]. The carotid artery compliance (CAC) was also calculated using the equation: CAC = ([Ds−Dd]/Dd)/(Ps−Pd). [Ps = systolic blood pressure (SBP), Pd = diastolic blood pressure (DBP)] [26].

### Real-time 3D and speckle tracking echocardiographic data

A 1–3 MHz 3D matrix phased array transducer (X3-1) was used to acquire 3D data. A wide-angle acquisition “full volume” mode was used. The full-volume acquisition was recorded with four consecutive cardiac cycles from the LV apical four-chamber view during an end-expiration breath hold with a mean frame rate of ≥ 20 frames/sec. To optimize the acquisition frame rate ≥ 20Hz, the depth and the sector width were adjusted to ensure that the entire LV cavity was included within the pyramidal volume [19,20].

**Fig 1:**
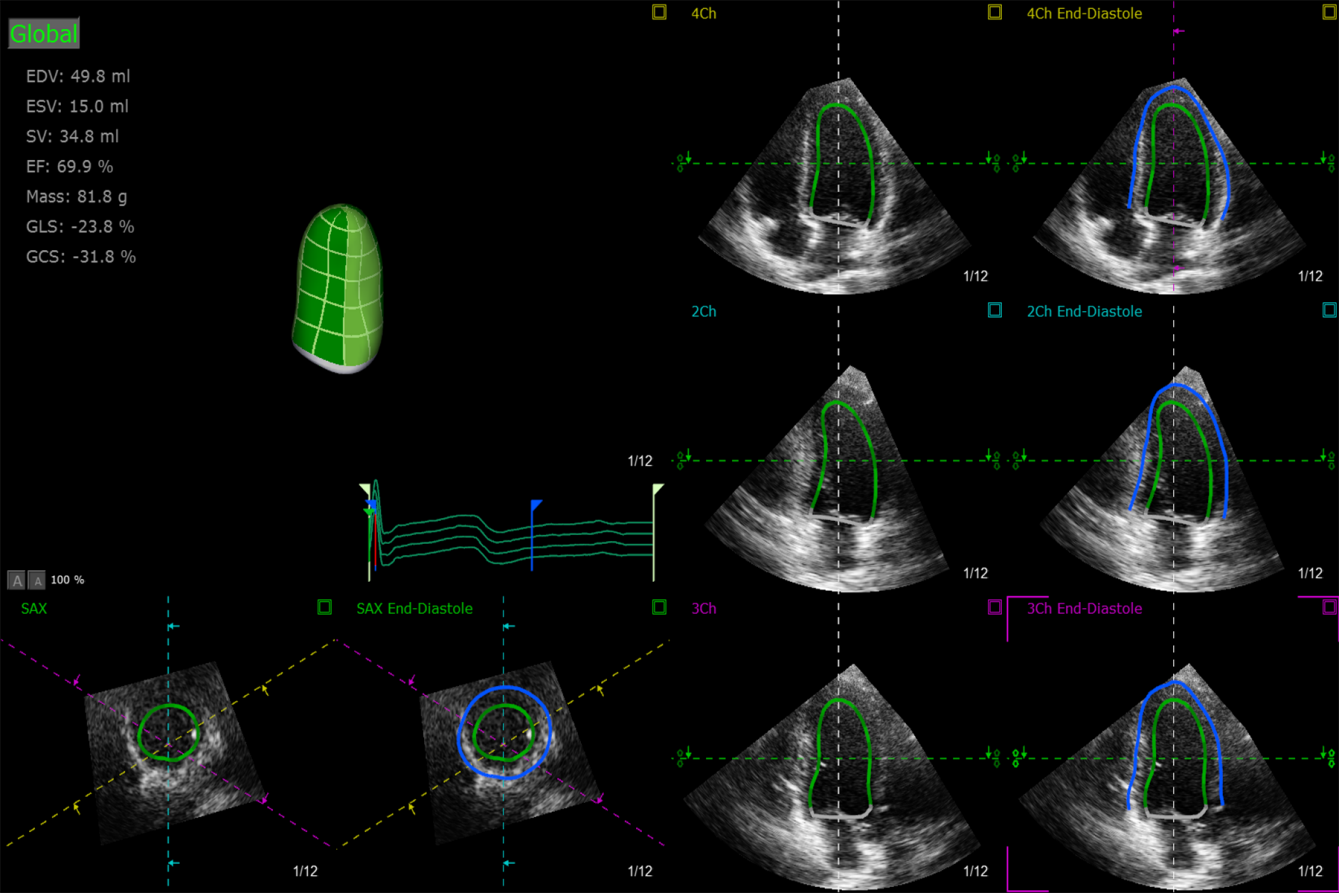
Offline analysis using 3-dimensional speckle tracking echocardiography. **(A)** Semi-automated endocardial border (*solid green line*) identification and tracking. **(B)** Semi-automated epicardium border (*solid blue line*) identification and tracking.

**Fig 2:**
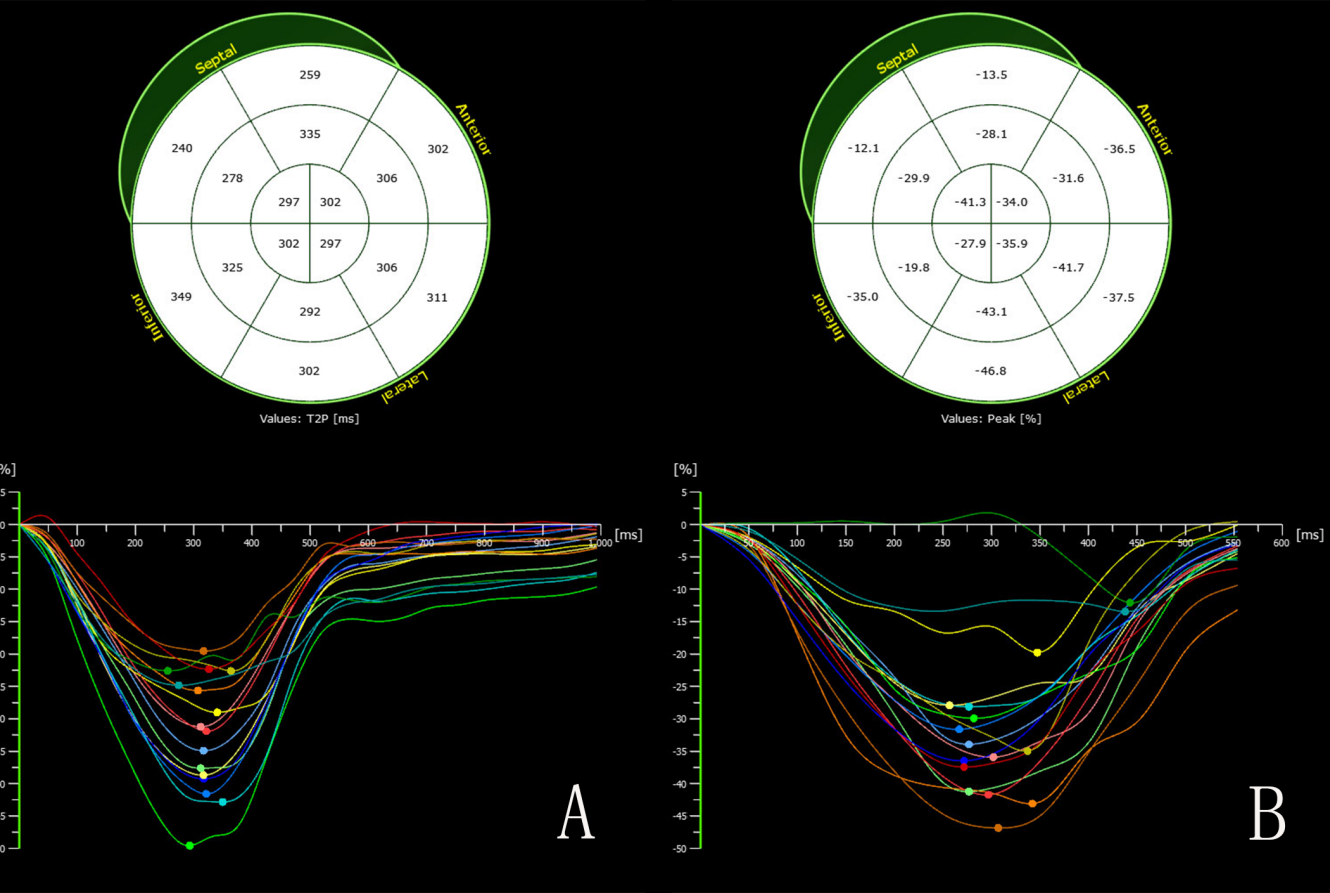
The 3-dimensional strain curves of the 16 segments of the LV in the controls (A) and in the obese subjects (B). The color-coded curves indicate the different myocardial segments of the left ventricle. The peak strain values of the several myocardial regions are decreased in the obese children compared with the controls.

Offline analysis of the data was performed using Tom-Tec analysis software (Tom Tec Imaging System GmbH, Unterschleissheim, Bayern, Germany). For the tracing of the endocardium and epicardium, the following four views were displayed: (1) the apical four-chamber view; (2) the apical three-chamber view; (3) the apical two-chamber view; and (4) the apex of the LV. The LV epicardium and endocardium were traced automatically (Fig 1), and the tracings were refined manually. Afterwards, the software automatically performed calculation of LV end-systolic volume (ESV) and end-diastolic volume (EDV), left ventricular ejection fraction (LVEF), left ventricular mass (LVM), global longitudinal strain (GLS), global radial strain (GRS), global circumferential strain (GCS) and global strain (GS) by 3D-STE [19,20]. A final 16-segment Bullseye map and curves of strain values was displayed (Fig 2). The left ventricular mass index (LVMI) was calculated as LVM divided by body surface area (BSA). The BSA was calculated using the equation: BSA = 0.0061 × height (cm) + 0.0128 × weight (kg) - 0.1529.

In order to have access to information that could identify individual participants during or after data collection, all the children were numbered with their information being recorded in an excel form.

### Inter- and Intra-observer reproducibility

The 3D data from 10 randomly selected subjects were analyzed twice at 1-week interval by one investigator for the intra-observer reproducibility. The interobserver reproducibility assessment was performed by analyzing data from 10 subjects who were chosen randomly by 2 independent investigators. The investigators were blinded to the other measurements.

### Statistical analysis

All the analyses were performed using SPSS version 17.0 software (SPSS, Inc., Chicago, IL, USA). The Shapiro-Wilk test was applied to ascertain normal distribution of parameters. Data from continuous variables were expressed as mean ± standard deviation. Differences in the normally distributed continuous data and categorical data were compared using the independent samples t-test and the chi-square test, respectively. The receiver operating characteristic (ROC) curve was also obtained to assess the sensitivity and specificity of the echocardiographic variables for assessing cardiovascular changes in the study group. Statistical tests were two-sided, and a *P* value < .05 was considered statistically significant. Inter- and intra-observer reproducibility values were evaluated using the intra-class correlation coefficient (ICC).

## Results

The demographic and clinical data of the study group, i.e., children with obesity, and of the control group are shown in Table 1. There was no significant difference in gender, height between the two groups; however, the study group had significantly increased weight, BMI, WC, HC, SBP, DBP, heart rate, age and subcutaneous fat thickness (P < 0.001 to 0.05).

**Table 1:** Clinical data for obese and normal control groups.

	Control	Obese	*P* values
Age (years)	11.49± 3.49	10.76 ± 2.72	< .05
Gender (male/female)	113/116	104/77	ns
Height (m)	1.44 ± 0.20	1.47 ± 0.16	ns
Weight (kg)	37.87 ±13.96	57.29 ± 19.25	< .001
BMI (kg/m^2^)	17.33 ± 2.58	25.67 ± 3.78	< .001
WC (cm)	63.77 ± 9.29	83.68 ± 11.66	< .001
HC (cm)	74.71 ± 13.02	88.90 ± 11.43	< .001
SBP (mm Hg)	101.41 ± 12.15	110.01 ± 12.91	< .001
DBP (mm Hg)	63.52 ± 9.58	69.71± 8.66	< .001
HR (bpm)	78.25± 10.87	81.83± 11.34	< .005
Subcutaneous fat (mm)	4.70 ± 1.88	12.62 ± 4.01	< .001

*BMI*, body mass index; *WC*, waist circumference; *HC*, hip circumference; *SBP*, systolic blood pressure; *DBP*, diastolic blood pressure; *HR*, heart rate.

The blood chemistry data are shown in Table 2. There was no significant difference in fasting glucose, triglyceride, and homocysteine levels between the two groups; however, the study group had significantly higher low-density lipoprotein cholesterol (LDL-C), total cholesterol, high-density lipoprotein cholesterol (HDL-C), CRP levels (P < 0.001 to< 0.05) than those of the normal control group (Table 2).

**Table 2:** Laboratory biochemistry data comparing obese and normal control groups.

	Control	Obese	*P* values
Total cholesterol (mmol/l)	4.01± 0.75	4.26 ± 0.88	< .05
LDL cholesterol (mmol/l)	2.16± 0.60	2.40 ± 0.65	< .01
HDL cholesterol (mmol/l)	1.37± 0.26	1.28 ± 0.29	< .01
TG (mmol/l)	1.46 ± 1.15	1.35± 0.91	ns
Fasting glucose (mmol/l)	5.05 ± 0.68	5.00 ± 0.53	ns
CRP (mg/l)	1.32 ± 1.43	3.13± 3.07	< .001
Homocysteine (μmol/l)	10.05 ±6.65	10.26 ± 8.57	ns

*TG*, triglycerides; *LDL*, low-density lipoprotein; *HDL*, high-density lipoprotein; *CRP*, C-reactive protein.

The conventional echocardiographic data are summarized in Table 3. There was no significant difference in LVEF, a’ between the study group and control group. However, the study group had increased LASD, IVSD, LVPWD, LVEDD, LVESD, LVEDV, LVESV, LVM, LVMI, E, A, e’, E/A, E /e’ and e’/a’ (P < 0.001 to 0.05) than those of the control group.

**Table 3:** Conventional echocardiographic data comparing obese and normal control groups.

	Control	Obese	P values
LASD (mm)	25.11 ± 3.73	28.57 ± 4.32	< .001
IVSD (mm)	6.20 ± 1.36	7.22 ± 1.48	< .001
LVPWD (mm)	6.35 ± 1.33	7.11± 1.39	< .001
LVEDD (mm)	39.29 ± 4.95	41.74 ± 4.52	< .001
LVESD (mm)	24.57 ± 3.81	26.32 ± 3.55	< .001
LVEDV (ml)	68.77 ± 20.55	76.74 ± 22.23	.001
LVESV (ml)	22.91 ± 10.30	27.47 ± 10.46	< .001
LVEF (%)	67.84 ± 5.80	67.11 ± 5.38	ns
E (cm/s)	108.56 ± 13.63	112.83 ± 14.91	< .05
A (cm/s)	57.19 ± 10.60	63.90 ± 12.67	< .001
E/A	1.95 ± 0.36	1.82 ± 0.40	< .01
e’(cm/s)	12.88 ± 1.41	11.99 ± 1.84	< .001
a’(cm/s)	6.93 ±6.27	7.13± 1.73	ns
e’/ a’	2.10 ± 0.49	1.75 ± 0.38	< .001
E/e’	8.49 ± 1.17	9.58 ± 1.63	< .001
LVM (g)	71.08 ± 29.84	99.32 ±43.05	< .001
LVMI (g/m^2^)	57.20 ± 14.21	66.15± 20.59	.001

*LASD*, left atrium end-systolic dimension; *IVSD*, interventricular septal end-diastolic dimension; *LVPWD*, left ventricular posterior wall end-diastolic dimension; *LVEDD*, left ventricular end-diastolic dimension; *LVESD*, left ventricular end-systolic dimension; *LVEDV*, left ventricular end-diastolic volume; *LVESV*, left ventricular end-systolic volume; *LVEF*, left ventricular ejection fraction; *E*, mitral E wave peak velocity; *A*, mitral A wave peak velocity; *e’*, peak e-wave velocity by tissue Doppler imaging; *a’*, peak a-wave velocity by tissue Doppler imaging; *LVM*, left ventricular mass; *LVMI*, left ventricular mass index.

Table 4 summarizes the vascular ultrasound data between the two groups. There was no significant difference in FMD and CAC between the study and the control groups. However, the study group had increased IMT than the control group (P < 0.001).

**Table 4:** Vascular ultrasound data comparing obese and normal control groups.

	Control	Obese	P values
FMD (%)	10.18 ±2.00	10.44 ± 1.82	ns
IMT (mm)	0.36 ± 0.08	0.46 ± 0.10	< .001
CAC (%/mm Hg)	0.15 ± 0.08	0.15 ± 0.06	ns

FMD, flow-mediated dilation; IMT, intima-media thickness; CAC, carotid artery compliance.

Table 5 shows the 3DSTE data for the 2 groups. Compared with the control group, the study group showed lower GLS, GCS, GRS and GS values (*P* < 0.001).

**Table 5:** Three-dimensional speckle tracking echocardiography global parameters comparing obese and normal control groups.

	Control	Obese	*P* values
GLS (%)	-21.12 ± 2.99	-17.57 ± 3.13	< .001
GRS (%)	41.56 ± 5.19	35.37 ± 5.58	< .001
GCS (%)	-28.36 ± 4.21	-24.34 ± 4.99	< .001
GS (%)	-33.48 ± 4.01	-30.10 ± 5.03	< .001

*GLS*, global longitudinal strain; *GRS*, global radial strain; *GCS*, global circumferential strain; *GS*, global strain.

Moderate inverse correlations were found between the BMI and GLS (r = 0.570, *P* <0.05, the BMI and GRS (r = -0.521, *P* <0.05), the BMI and GCS (r = 0.380, *P* <0.05), the LVM and GLS (r = 0.411, *P* <0.05), the LVM and GRS (r = -0.369, *P* <0.05), and the LVM and GCS (r = 0.268, *P* <0.05).

**Fig 3:**
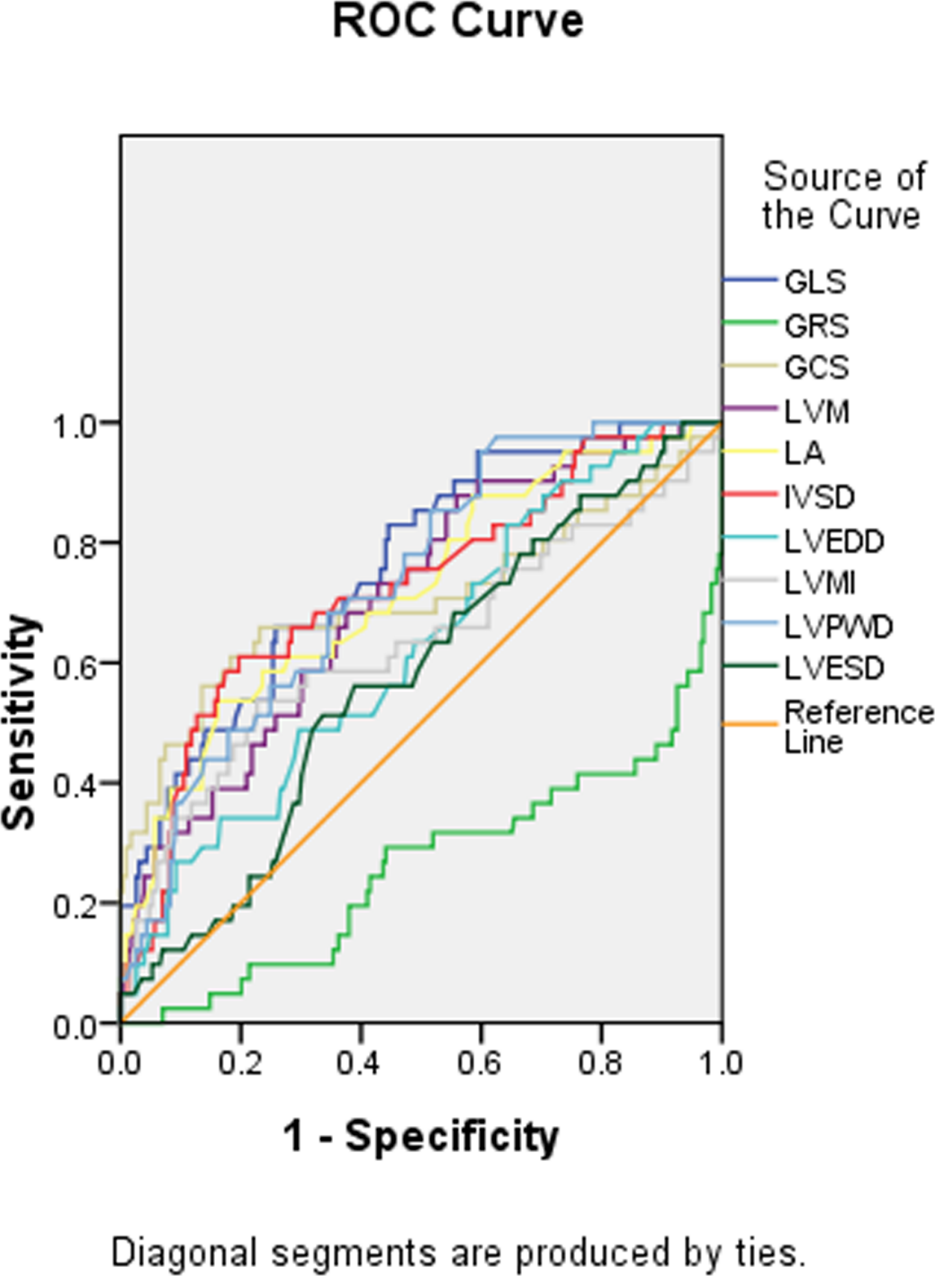
The areas under the ROC for the echocardiographic and vascular ultrasound variables.

In Table 6, the ROC for the statistically significant echocardiographic variables showed that the range of areas under the ROC curves varied from 0.76 (GLS), 0.74 (GRS), 0.72 (LASD), to 0.58 (LVESD)(Fig 3).

**Table 6:** Area under ROC curve for the echocardiographic variables.

Test Result Variable(s)	Area	Asymptotic 95% Confidence Interval	
		Lower Bound	Upper Bound
GLS	0.76	0.69	0.84
GRS	0.74	0.65	0.83
GCS	0.71	0.60	0.81
LVM	0.71	0.62	0.79
IVSD	0.72	0.64	0.81
LASD	0.72	0.63	0.81
LVEDD	0.62	0.53	0.71
LVMI	0.63	0.52	0.74
LVPWD	0.73	0.66	0.81
LVESD	0.58	0.48	0.67

*GLS*, global longitudinal strain; *GRS*, global radial strain; *GCS*, global strain circumferential; *LVM*, left ventricular mass; *IVSD*, interventricular septal end-diastolic dimension; *LASD*, left atrium end-systolic dimension; *LVEDD*, left ventricular end-diastolic dimension; *LVMI*, left ventricular mass index; *LVPWD*, left ventricular posterior wall end-diastolic dimension; *LVESD*, left ventricular end-systolic dimension; *IMT*, intima-media thickness; *FMD*, flow-mediated dilation; *LVEF*, left ventricular ejection fraction; *CAC*, carotid artery compliance

### Inter-observer and Intra-observer reproducibility

Inter-observer agreement assessed by the ICC was 0.84, 0.83, 0.76 and 0.76 for GLS, GRS, GCS, and GS, respectively (Table 7), whereas intra-observer agreement ICC 0.78, 0.82, 0.79 and 0.87 for GLS, GRS, GCS, and GS (Table 8).

**Table 7:** Inter-observer variability of GLS, GCS, GRS, and GS by 3D-STE.

	Observer1	Observer2	ICC
GLS (%)	-18.46±2.95	-18.68±2.93	0.83
GRS (%)	38.75±7.01	37.01±6.02	0.84
GCS (%)	-27.45±5.43	-25.37±4.96	0.76
GS (%)	-33.08±5.55	-31.40±4.13	0.76

*ICC*, intraclass correlation coefficient.

**Table 8:** Intra-observer variability of GLS, GCS, GRS, and GS by 3D-STE.

	Observer1	Observer2	ICC
GLS (%)	-19.63±3.83	-19.52±3.30	0.78
GRS (%)	41.64±6.17	42.17±4.91	0.82
GCS (%)	-30.42±3.92	-31.09±3.29	0.79
GS (%)	-34.84±3.25	-35.63±3.44	0.87

*ICC*, intraclass correlation coefficient.

## Discussion

We evaluated the association between obesity and cardiovascular changes using clinical, biochemical, vascular ultrasound, and echocardiographic (2D, Doppler, and RT3DE/3DSTE) variables. In addition to an increase in weight, adiposity variables, our study showed significant differences in blood pressures, cholesterol levels, IMT, LV size, wall thickness, mass, and systolic and diastolic function in children with obesity. In this cohort, there was no difference in vascular ultrasound variables (FMD and CAC). In contrast, the 3D strain variables were significantly diminished in obese children compared with those in the normal control group. Finally, the ROC analysis showed that 3D LV strain variables, LASD are the most sensitive variables to detect LV changes associated with obesity in children.

Based on the strengths and limitations of the conventional echocardiographic measurement of LV function [27], we previously validated the feasibility, reproducibility, maturational changes, and normal ranges of 3D strain and other variables of the LV in normal children using RT3DE and 3DSTI technologies [19,20]. In this study, we applied these LV 3D strain analyses to evaluate children with obesity.

There are a few findings in this study worthy of discussion. First, the age of our study group was 11.5 ± 3.5 years, representing early cardiovascular changes associated with obesity. Obesity can lead to cardiac output increase and high LV wall tension, which may result in thickening of LV wall to compensate LV wall tension [28]. On the other hand, LV hypertrophy adapts to the enlargement of LV cavities and impairment of diastolic function while global systolic function is preserved. Previous epidemiological studies [4–6,8–10] have shown that obesity is associated with cardiovascular alterations, including atherosclerotic changes in autopsies, subclinical structural and functional alterations by vascular ultrasound studies, and other risk factors for CVD such as hypertension, dyslipidemia, insulin resistance, and metabolic syndrome in children and adolescents. On the other hand, obesity, with or without hypertension, can also cause left ventricular remodeling, e.g. increased left ventricular chamber dimensions, volumes, wall thickness, and mass. Our study results suggested that LV adaptation and remodeling may occur at a young age in obese children and precede other cardiovascular changes that have been shown in adolescents and young adults with obesity or other CVD risk factors.

Secondly, we demonstrated that the LV 3D strain variables decreased in obese children and that these variables are sensitive for detecting myocardial functional alterations. The reasons for these findings are likely related to adiposity and myocardial abnormality in obesity. Fat accumulation causes irregular adipose tissue aggregation between the myocardial cells, pressure increase, myocardial cell atrophy, and cardiac dysfunction [29]. Furthermore, the hormones and proinflammatory cytokines that the adipose cells secrete may affect myocardial remodeling [30], i.e. LV enlargement, hypertrophy, diastolic dysfunction, and 3D strain abnormalities but preserved LVEF [31–35]. Although our obese subjects had a normal LVEF, they demonstrated evidence of impaired systolic function manifested by decreased LV systolic strain. The abnormalities in cardiac function observed in our subjects likely involve myocardial fibers which affect myocardial deformation. These results suggest that conventional measures of cardiac function, e.g. LVEF, may not provide sensitive assessment of early alteration of cardiac dysfunction, while 3D-STE has the capabilities.

Thirdly, our data showed that GLS, GRS, GCS, and GS were significantly decreased in obese children without comorbidities. These findings are consistent with results from previous studies that showed similar differences using different methods and measurements for LV strain variables [5,8,36], whereas they differ from others that showed there was no difference in LV strain variables in obese children [6,37]. The heterogeneity in the results from the previous studies can be explained as follows: (1) STE is based on tracking and measurement of tissue displacement. Whereas 2DSTE tracks speckle movement on 2D planes, cardiac movement is 3D. As a result, 2DSTE tracks the projections of the speckles moving out of the plane, which are less than the real distance between the speckles. In contrast, 3DSTE is free of geometric assumptions and speckles moving out of the scanning plane. Therefore, 3DSTE is likely more accurate than 2DSTE for evaluating LV strain variables. (2) LV strain results calculated using different echocardiographic systems, software, and algorisms can also cause variabilities in strain measurements, as with previous studies using different echocardiographic systems and tracking algorithms [38]. (3) Age, gender, and ethnicity may also contribute to the variable LV strain results in obese children in the literature.

Finally, among the CVD risk factors, BMI is found to be an independent predictor of worsening LV systolic and diastolic function [32]. Our study showed that 3D LV strain variables appeared to be more closely related to BMI than to LVM as was reported in the previous studies, e.g. BMI is more closely associated with a decrease in GLS [37,39], GCS, or GRS in our study. Left ventricular hypertrophy induced by increased wall stress can damage the subendocardial myocardial fibers, which are responsible for regional myocardial function [40]. In our study, cholesterol levels were significantly higher in the obese group than in the control group, which are similar to the findings from a previous study [41–43], whereas there was no difference in TG, fasting glucose, and homocysteine levels between the obese and the control group. In our study, IMT was significantly higher in the obese group than in the control group, which are similar to the findings from a previous study [42], however there was no significant difference in FMD, and CAC among obese and control subjects, unlike some of the previous studies [26,44]. Again, the differences may be explained by the younger age, the ethnicity of the subjects, and other variables in this study.

### Limitations

There are limitations in this study. First, all subjects were Chinese in ethnicity. The data and results may be different for other ethnic groups. There were incomplete data in demographic, echocardiography and blood chemistry, due to the concern of the subject and their families. Further research is necessary to assess the variability of 3D STE for the evaluation of LV 3D strain in other ethnic population. Second, strain measurements among different vendors had inter-vendor variability. The use of a single vendor is appropriate for early research applications, but clinical applications in large populations across multiple imaging platforms need standardization. Finally, 3D STE is highly dependent on image quality, especially endocardial boundary delineation, and its low frame rate may lead to miscorrelation among frames and affect strain data accuracy. Technology will likely improve in temporal resolution, sector size and width, and more modality independent and automated 3D speckle tracking echocardiographic computational algorithms.

## Conclusions

LV 3D strain variables by 3DSTE are decreased in young obese children. LV 3D strain variables are more sensitive than other conventional echocardiographic and vascular ultrasound variables in detecting early cardiovascular abnormalities in children with obesity.

## Supporting information

S1 Table Control and obese group data. (XLS)

## Abbreviations

2D: two-dimensional
3D: three-dimensional
A: late transmitral inflow velocities
a’: late diastolic mitral annular peak velocities
BMI: body mass index
BSA: body surface area
CAC: carotid artery compliance
CRP: C-reactive protein
CVD: cardiovascular disease
DBP: diastolic blood pressure
E: early transmitral inflow velocities
e’: early diastolic mitral annular peak velocities
EDV: end-diastolic volume
ESV: end-systolic volume
FMD: flow-mediated dilation
GCS: global circumferential strain
GLS: global longitudinal strain
GRS: global radial strain
GS: global strain
HC: hip circumference
HDL-C: high-density lipoprotein cholesterol
ICC: intra-class correlation coefficient
IMT: intima-media thickness
IVSD: interventricular septal end-diastolic dimension
LASD: left atrium end-systolic dimension
LDL-C: low-density lipoprotein cholesterol
LV: left ventricle
LVEDD: left ventricular end-diastolic dimension
LVESD: left ventricular end-systolic dimension
LVEF: left ventricular ejection fraction
LVM: left ventricular mass
LVMI: left ventricular mass index
LVPWD: left ventricular posterior wall end-diastolic dimension
ROC: receiver operating characteristic
SBP: systolic blood pressure
STE: speckle tracking echocardiography
TDI: tissue doppler imaging
TG: triglycerides
WC: waist circumference

## References

[1] Sorof J, Daniels S. Obesity hypertension in children: a problem of epidemic proportions. Hypertension 2002;40:441–7. 12364344 10.1161/01.hyp.0000032940.33466.12

[2] Alpert MA. Obesity cardiomyopathy: pathophysiology and evolution of the clinical syndrome. Am J Med Sci 2001;321:225–36. 11307864 10.1097/00000441-200104000-00003

[3] Kenchaiah S, Evans JC, Levy D, Wilson PW, Benjamin EJ, Larson MG, et al Obesity and the risk of heart failure. N Engl J Med 2002;347:305–33. 10.1056/NEJMoa020245 12151467

[4] Emerging Risk Factors Collaboration, Wormser D, Kaptoge S, Di Angelantonio E, Wood AM, Pennells L, et al Separate and combined associations of body-mass index and abdominal adiposity with cardiovascular disease: collaborative analysis of 58 prospective studies. Lancet 2011;377(9771):1085–95. 10.1016/S0140-6736(11)60105-0 21397319 PMC3145074

[5] Di Salvo G, Pacileo G, Del Giudice EM, Natale F, Limongelli G, Verrengia M, et al Abnormal myocardial deformation properties in obese, non-hypertensive children: an ambulatory blood pressure monitoring, standard echocardiographic, and strain rate imaging study. Eur Heart J 2006;27:2689–95. 10.1093/eurheartj/ehl163 16905554

[6] Labombarda F, Zangl E, Dugue AE, Bougle D, Pellissier A, Ribault V, et al Alterations of left ventricular myocardial strain in obese children. Eur Heart J Cardiovasc Imaging 2013;14:668–76. 10.1093/ehjci/jes238 23161790

[7] Li X, Li S, Ulusoy E, Chen W, Srinivasan SR, Berenson GS. Childhood adiposity as a predictor of cardiac mass in adulthood: the Bogalusa Heart Study. Circulation 2004;110:3488–92. 10.1161/01.CIR.0000149713.48317.27 15557363

[8] Saltijeral A, Isla LP, Pérez-Rodríguez O, Rueda S, Fernandez-Golfin C, Almeria C et al Early myocardial deformation changes associated to isolated obesity: a study based on 3D-wall motion tracking analysis. Obesity (Silver Spring) 2011;19:2268–73. 21720437 10.1038/oby.2011.157

[9] Körner A, Wiegand S, Hungele A, Tuschy S, Otto KP, l'Allemand-Jander D et al Longitudinal multicenter analysis on the course of glucose metabolism in obese children. Int J Obes (Lond) 2013;37:931–6. 23032406 10.1038/ijo.2012.163

[10] Wong CY, O’Moore-Sullivan T, Leano R, Byrne N, Beller E, Marwick TH. Alterations of left ventricular myocardial characteristics associated with obesity. Circulation 2004;110:3081–7. 10.1161/01.CIR.0000147184.13872.0F 15520317

[11] Gorscan J III, Strum DP, Mandarino WA, Gulati VK, Pinsky MR. Quantitative assessment of alterations in regional left ventricular contractility with color-coded tissue Doppler echocardiography. Comparison with sonomicrometry and pressure-volume relations. Circulation 1997;95: 2423–33. 9170406 10.1161/01.cir.95.10.2423

[12] Eidem BW, McMahon CJ, Cohen RR, Wu J, Finkelshteyn I, Kovalchin JP, et al Impact of cardiac growth on Doppler tissue imaging velocities: a study in healthy children. J Am Soc Echocardiogr 2004;17:212–21. 10.1016/j.echo.2003.12.005 14981417

[13] Korinek J, Wang J, Sengupta PP, Miyazaki C, Kjaergaard J, McMahon E, et al Two-dimensional strain—a Doppler-independent ultrasound method for quantitation of regional deformation: validation in vitro and in vivo. J Am Soc Echocardiogr 2005;18:1247–53. 10.1016/j.echo.2005.03.024 16376750

[14] Amunsen BH, Helle-Valle T, Edvardsen T, Torp H, Crosby J, Lyseggen E, et al Noninvasive myocardial strain measurement by speckle tracking echocardiography: validation against sonomicrometry and tagged magnetic resonance imaging. J Am Coll Cardiol 2006;47:789–93. 10.1016/j.jacc.2005.10.040 16487846

[15] Young AA, Axel L, Dougherty L, Bogen DK, Parenteau CS. Validation of tagging with MR imaging to estimate material deformation. Radiology 1993;188:101–8. 10.1148/radiology.188.1.8511281 8511281

[16] Moore CC, Lugo-Olivieri CH, McVeigh ER, Zerhouni EA. Three-dimensional systolic strain patterns in the normal human left ventricle: characterization with tagged MR imaging. Radiology 2000;214:453–66. 10.1148/radiology.214.2.r00fe17453 10671594 PMC2396279

[17] Marcus KA, Mavinkurve-Groothuis AM, Barends M, van Dijk A, Feuth T, de Korte C, et al Reference values for myocardial two-dimensional strain echocardiography in a healthy pediatric and young adult cohort. J Am Soc Echocardiogr 2011;24:625–36. 10.1016/j.echo.2011.01.021 21392941

[18] Lang RM, Badano LP, Mor-Avi V, Afilalo J, Armstrong A et al Recommendations for cardiac chamber quantification by echocardiography in adults: an update from the American Society of Echocardiography and the European Association of cardiovascular imaging. J Am Soc Echocardiogr. 2015 1;28(1):1–39. 10.1016/j.echo.2014.10.003 25559473

[19] Zhang L, Gao J, Xie M, Yin P, Liu W, Li Y.et al Left ventricular three-dimensional global systolic strain by real-time three-dimensional speckle-tracking in children: feasibility, reproducibility, maturational changes, and normal ranges. J Am Soc Echocardiogr 2013;26(8):853–9. 10.1016/j.echo.2013.05.002 23791113

[20] Zhang L, Zhang J, Han W, Gao J, He L, Yang Y, et al Three-dimensional rotation, twist and torsion analyses using real-time 3D speckle tracking imaging: feasibility, reproducibility, and normal ranges in pediatric population. PLoS ONE 2016;11(7):e0158679 10.1371/journal.pone.0158679 27427968 PMC4948847

[21] Group of China Obesity Task Force. [Body mass index reference norm for screening overweight and obesity in Chinese children and adolescents]. [Article in Chinese]. Zhonghua Liu Xing Bing Xue Za Zhi [Chinese Journal of Epidemiology] 2004;25(2):97–102. 15132858

[22] National High Blood Pressure Education Program Working Group on High Blood Pressure in Children and Adolescents. The fourth report on diagnosis, evaluation, and treatment of high blood pressure in children and adolescents. Pediatrics 2004;114:555–576. 15286277

[23] Refsum H, Ueland PM, Svardal AM. Fully automated fluorescence assay for determining total homocysteine in plasma. Clin Chem 1989;35:1921–7. 2776317

[24] Oliveira OP, Araujo Júnior E, Lima JW, Salustiano EM, Ruano R, et al Flow-mediated dilation of brachial artery and endothelial dysfunction in pregnant women with preeclampsia: a case control study. Minerva Ginecol 2015;67(4):307–313. 25476264

[25] Juonala M, Viikari JS, Rönnemaa T, Taittonen L, Marniemi J, Raitakari OT. Childhood C-reactive protein in predicting CRP and carotid intima-media thickness in adulthood: The Cardiovascular Risk in Young Finns Study. Arterioscler Thromb Vasc Biol 2006;26:1883–8. 10.1161/01.ATV.0000228818.11968.7a 16728658

[26] Juonala M, Viikari JS, Rönnemaa T, Taittonen L, Marniemi J, Raitakari OT. Risk factors identified in childhood and decreased carotid artery elasticity in adulthood: The Cardiovascular Risk in Young Finns Study. Circulation 2005;112(10):1486–93. 10.1161/CIRCULATIONAHA.104.502161 16129802

[27] Pacileo G, Di Salvo G, Limongelli G, Miele T, Calabrò R. Echocardiography in congenital heart disease: usefulness, limits and new techniques. J Cardiovasc Med (Hagerstown) 2007;8:17–22. 17255811 10.2459/01.JCM.0000247430.36581.c2

[28] Wang Q, Gao Y, Tan K, Li P. Subclinical impairment of left ventricular function in diabetic patients with or without obesity: A study based on three-dimensional speckle tracking echocardiography. Herz 2015;40(3):260–268. 25491664 10.1007/s00059-014-4186-y

[29] Spain DM, Cathcart RT. Heart block caused by fat infiltration of the interventricular septum (cor adiposum). Am Heart J 1946;32(5):659–64. 20274725 10.1016/0002-8703(46)90674-6

[30] Dervan JP, Ilercil A, Kane PB, Anagnostopoulos C. Fatty infiltration: another restrictive cardiomyopathic pattern. Cathet Cardiovasc Diagn 1991:22(3):184–9. 2013082 10.1002/ccd.1810220307

[31] Galinier M, Pathak A, Roncalli J, Massabuau P. [Obesity and cardiac failure.] [Article in French]. Arch Mal Coeur Vaiss 2005;98(1):39–45. 15724418

[32] Peterson LR, Waggoner AD, Schechtman KB, Meyer T, Gropler RJ, Barzilai B, et al Alterations in left ventricular structure and function in young healthy obese women: assessment by echocardiography and tissue Doppler imaging. JACC 2004;43(8):1399–404. 10.1016/j.jacc.2003.10.062 15093874

[33] Rodriguez L, Garcia M, Ares M, Griffin BP, Nakatani S, Thomas JD. Assessment of mitral annular dynamics during diastole by Doppler tissue imaging: comparsion with mitral Doppler inflow in subjects without heart disease and in patients with left ventricular hypertrophy. Am Heart J 1996;131(5):982–7. 8615320 10.1016/s0002-8703(96)90183-0

[34] Luis SA, Yamada A, Khandheria BK, Speranza V, Benjamin A, Ischenko M, et al Use of three-dimensional speckle-tracking echocardiography for quantitative assessment of global left ventricular function: a comparative study to three-dimensional echocardiography. J Am Soc Echocardiogr 2014;27:285–91. 10.1016/j.echo.2013.11.002 24325960

[35] Kaku K, Takeuchi M, Tsang W, Takigiku K, Yasukochi S, Patel AR et al Age-related normal range of left ventricular strain and torsion using three-dimensional speckle-tracking echocardiography. J Am Soc Echocardiogr 2014;27:55–64. 10.1016/j.echo.2013.10.002 24238753

[36] Mangner N, Scheuermann K, Winzer E, Wagner I, Hoellriegel R, Sandri M, et al Childhood obesity: impact on cardiac geometry and function. JACC Cardiovasc Imaging 2014;7(12):1198–205. 10.1016/j.jcmg.2014.08.006 25306542

[37] Kibar AE, Pac FA, Ece İ, Oflaz MB, Ballı Ş, Bas VN et al Effect of obesity on left ventricular longitudinal myocardial strain by speckle tracking echocardiography in children and adolescents. Balkan Med J 2015;32(1):56–63. 10.5152/balkanmedj.2015.15136 25759773 PMC4342139

[38] Gayat E, Ahmad H, Weinert L, Lang RM, Mor-Avi V. Reproducibility and inter-vendor variability of left ventricular deformation measurements by three-dimensional speckle-tracking echocardiography. J Am Soc Echocardiogr 2011;24(8):878–85. 10.1016/j.echo.2011.04.016 21645991

[39] Barbosa JA, Mota CC, Simões E Silva AC, Nunes Mdo C, Barbosa MM. Assessing pre-clinical ventricular dysfunction in obese children and adolescents: the value of speckle tracking imaging. Eur Heart J Cardiovasc Imaging 2013;14(5):882–9. 23291394 10.1093/ehjci/jes294

[40] Lorch SM, Sharkey A. Myocardial velocity, strain, and strain rate abnormalities in healthy obese children. J Cardiometab Syndr 2007;2:30–4. 17684447 10.1111/j.1559-4564.2007.06001.x

[41] Vitarelli A, Martino F, Capotosto L, Martino E, Colantoni C, Ashurov R, et al Early myocardial deformation changes in hypercholesterolemic and obese children and adolescents. A 2D and 3D speckle tracking echocardiography study. Medicine (Baltimore) 2014;93(12):e71–e71. 25211047 10.1097/MD.0000000000000071 PMC4616267

[42] Simşek E, Balta H, Balta Z, Dallar Y. Childhood obesity-related cardiovascular risk factors and carotid intima-media thickness. Turk J Pediatr 2010;52(6):602–11. 21428192

[43] Whincup PH, Gilg JA, Donald AE, Katterhorn M, Oliver C, Cook DG, et al Arterial distensibility in adolescents: The influence of adiposity, the metabolic syndrome, and classic risk factors. Circulation 2005;112 (12):1789–1797. 10.1161/CIRCULATIONAHA.104.532663 16172286

[44] Kozakova M, Morizzo C, Bianchi V, Marchetti S, Federico G, Palombo C. Hemodynamic overload and intra-abdominal adiposity in obese children: Relationships with cardiovascular structure and function. Nutr Metab Cardiovasc Dis 2015,26(1):60–66. 10.1016/j.numecd.2015.10.002 26643211

